# Serum Albumin Levels Are Associated with Total Brain and Hippocampal Volume but Not with White Matter Lesion Volume in Older Japanese Adults Without Cognitive Decline: A Cross-Sectional Study

**DOI:** 10.3390/nu18101520

**Published:** 2026-05-10

**Authors:** Yuta Usui, Moeko Noguchi-Shinohara, Makoto Mori, Shutaro Shibata, Taro Ozaki, Ayano Shima, Yasuyuki Taki, Kazuhiro Uchida, Takanori Honda, Jun Hata, Tomoyuki Ohara, Tatsuya Mikami, Tetsuya Maeda, Masaru Mimura, Kenji Nakashima, Jun-ichi Iga, Minoru Takebayashi, Toshiharu Ninomiya, Kenjiro Ono, on behalf of The Japan Prospective Studies Collaboration for Aging and Dementia (JPSC-AD) Study Group

**Affiliations:** 1 Department of Neurology, Kanazawa University Graduate School of Medical Sciences, 13-1 Takaramachi, Kanazawa 920-8640, Japan; 2 Department of Aging Research and Geriatric Medicine, Institute of Development, Aging and Cancer, Tohoku University, 4-1 Seiryo-Cho, Aoba-ku, Sendai 980-8575, Japan; 3 Department of Nutritional Sciences, Nakamura Gakuen University, 5-7-1 Befu, Jonan-ku, Fukuoka 814-0198, Japan; 4 Department of Epidemiology and Public Health, Graduate School of Medical Sciences, Kyushu University, 3-1-1 Maidashi, Higashi-ku, Fukuoka 812-8582, Japan; 5 Center for Cohort Studies, Graduate School of Medical Sciences, Kyushu University, 3-1-1 Maidashi, Higashi-ku, Fukuoka 812-8582, Japan; 6 Department of Health Care Administration and Management, Graduate School of Medical Sciences, Kyushu University, 3-1-1 Maidashi, Higashi-ku, Fukuoka 812-8582, Japan; 7 Department of Neuropsychiatry, Graduate School of Medical Sciences, Kyushu University, 3-1-1 Maidashi, Higashi-ku, Fukuoka 812-8582, Japan; 8 Department of Preemptive Medicine, Innovation Center for Health Promotion, Graduate School of Medicine, Hirosaki University, 5 Zaifu-cho, Hirosaki 036-8562, Japan; 9 Division of Neurology and Gerontology, Department of Internal Medicine, School of Medicine, Iwate Medical University, 2-1-1 Idaidori, Yahaba-cho, Iwate 028-3695, Japan; 10 Center for Preventive Medicine, Keio University, Azabudai Hills Mori JP Tower 7th Floor, 1-1-1 Azabudai, Minato-ku, Tokyo 106-0041, Japan; 11 NHO Matsue Medical Center, 5-8-31 Kaminogi, Matsue 690-8556, Japan; 12 Department of Neuropsychiatry, Ehime University Graduate School of Medicine, Shitsukawa, Toon, Ehime 791-0295, Japan; 13 Department of Psychiatry and Neuroscience, Center for Metabolic Regulation of Healthy Aging, Faculty of Life Sciences, Kumamoto University, 1-1-1 Honjo, Chuo-ku, Kumamoto 860-8556, Japan

**Keywords:** hypoalbuminemia, magnetic resonance imaging, hippocampus, frailty, cross-sectional studies

## Abstract

**Background/Objectives**: Serum albumin has antioxidant, anti-inflammatory, and antithrombotic properties and reflects nutritional status. Hypoalbuminemia is linked to cognitive decline and frailty. However, the relationship between serum albumin levels and brain structural changes in older adults remains unclear. We aimed to examine the associations between serum albumin levels and total brain, hippocampal, and white matter lesion volumes in cognitively normal, community-dwelling older Japanese adults, accounting for frailty status. **Methods**: In this cross-sectional study, 7266 participants aged ≥65 years without cognitive decline were included. Serum albumin levels, maximum handgrip strength, and usual gait speed were measured in all participants. Brain magnetic resonance imaging scans were used to evaluate total brain, hippocampal, and white matter lesion volumes. **Results**: Lower serum albumin levels were significantly associated with smaller total brain and hippocampal volumes after multivariable adjustment (both *p* for trend < 0.001; partial *η^2^* = 0.005), but not with white matter lesion volumes (*p* for trend = 0.24; partial *η^2^* = 0.001). In subgroup analyses stratified by frailty status, no significant heterogeneity in the associations between serum albumin levels and each brain volume was observed between groups defined by maximum handgrip strength or usual gait speed. **Conclusions**: Lower serum albumin levels are associated with smaller total brain and hippocampal volumes in cognitively normal, community-dwelling older Japanese adults, irrespective of frailty status. Serum albumin may serve as a clinically accessible marker of nutritional conditions in relation to these brain structures in older adults.

## 1. Introduction

With global population aging, the prevalence of dementia is rapidly increasing, and the associated medical and caregiving burden has become a major societal challenge [1]. Effective primary prevention of dementia requires identification of risk factors during the preclinical stage and timely intervention targeting modifiable factors. Several epidemiological studies have demonstrated that low serum albumin levels are associated with cognitive decline [2,3,4,5,6]. However, the relevance of serum albumin to brain health may extend beyond its role as a general nutritional marker. As the most abundant plasma protein, serum albumin reflects nutritional status and contributes to systemic homeostasis through its antioxidant, anti-inflammatory, and antithrombotic properties [7]. Experimental studies have suggested that serum albumin may exert neuroprotective effects in the brain, including protection against neuronal injury, oxidative stress, and neuronal apoptosis [8,9]. Limited human magnetic resonance imaging (MRI) studies have also linked serum albumin to brain structure [10,11]. In addition, previous studies have reported that frailty and prefrailty were associated with lower brain volume and greater white matter abnormalities in older adults [12,13]. However, few studies have directly examined the relationship between serum albumin levels and MRI-based brain structural features in community-dwelling older adults with normal cognitive function. Given that serum albumin is also associated with physical frailty, it is possible that the relationship between albumin and brain structure differs according to underlying physical reserve.

The Japan Prospective Studies Collaboration for Aging and Dementia (JPSC-AD) is an ongoing observational study of dementia in community-dwelling older adults across eight research sites. At baseline, approximately 10,000 participants underwent brain MRI scans and dietary assessments. Details of the survey have been reported previously [14]. In the present study, we aimed to determine the relationships between serum albumin levels and total brain volume (TBV), hippocampal volume (HV), and white matter lesion volume (WMLV) using brain MRI data from community-dwelling older Japanese adults with normal cognitive function, while accounting for frailty status. We hypothesized that lower serum albumin levels would be associated with less favorable MRI-based brain structural characteristics. We also examined whether these associations differed according to frailty status. Clarifying these associations may help to better define the clinical relevance of serum albumin in later life.

## 2. Materials and Methods

### 2.1. Study Population

**Figure 1:**
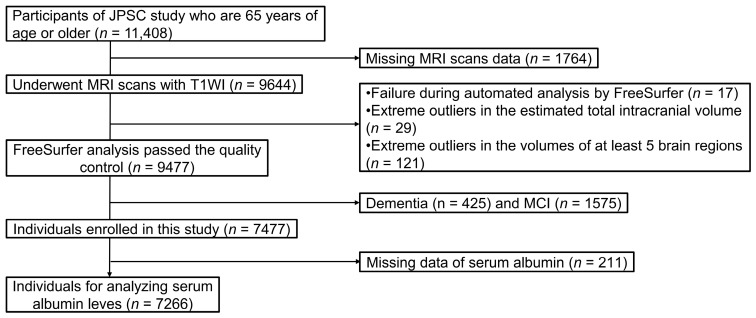
Flowchart of the participant selection. JPSC-AD, Japan Prospective Studies Collaboration for Aging and Dementia; MCI, mild cognitive impairment; MRI, magnetic resonance imaging; T1WI, T1-weighted imaging.

This cross-sectional study used baseline data from the JPSC-AD conducted between 2016 and 2018. Among the 11,408 participants aged 65 years or older from eight research sites who provided informed consent, 9644 underwent MRI scanning with three-dimensional T1-weighted imaging. After excluding 17 participants in whom automated processing with FreeSurfer failed, 29 with extreme outlier values for estimated total intracranial volume (eTIV), 121 with extreme outlier values in at least five regional brain volumes, 2000 individuals diagnosed with dementia (*n* = 425) or mild cognitive impairment (*n* = 1575) at baseline, and 211 lacking serum albumin measurements, the remaining 7266 participants were included in the analysis (Figure 1).

### 2.2. Ethical Approval and Informed Consent

The study protocol was approved by the Kyushu University Institutional Review Board for clinical research (Approval Number 24116, 23 October 2025). The study procedures conformed to the ethical standards of the Declaration of Helsinki. Written informed consent was obtained from all participants.

### 2.3. MRI Analysis

Brain MRI was performed using T1-weighted imaging in accordance with the Alzheimer’s Disease Neuroimaging Initiative protocol [15]. The MRI protocol was standardized across the participating research sites, and inter-scanner differences were corrected using phantom-based calibration. FreeSurfer (version 7.0; http://surfer.nmr.mgh.harvard.edu) was used to segment cortical and subcortical regions and derive volumetric measures, including TBV, HV, WMLV, and eTIV. TBV was derived from segmented brain volumes after excluding ventricular volumes. Cortical regions were parcellated according to the Desikan–Killiany atlas [16]. To adjust for individual differences in intracranial volume, each brain-volume measure was normalized to eTIV and analyzed as TBV/eTIV, HV/eTIV, and WMLV/eTIV (%).

### 2.4. Measurements of Serum Albumin Levels

Serum albumin levels were measured at the LSI Medience Corporation central laboratory (Tokyo, Japan) using the modified bromocresol purple dye-binding method in samples collected between 2016 and 2018. Supplementary Table S1 summarizes the serum albumin measurement procedures. Serum albumin levels were first classified using the clinical cutoff of <3.5 g/dL (hypoalbuminemia) [17]. Participants with albumin ≥3.5 g/dL were further divided into quartiles to allow stepwise evaluation above the clinical cutoff: 3.5–4.2, 4.2–4.3, 4.3–4.6, and ≥4.6 g/dL. For subgroup analyses with frailty-related indicators, serum albumin was dichotomized as <4.2 vs. ≥4.2 g/dL based on the primary categorical analysis. For sensitivity analyses, albumin levels were further classified into quintiles to assess the robustness of the findings, as follows: <4.1, 4.1–4.2, 4.3, 4.4–4.5, and ≥4.6 g/dL.

### 2.5. Assessment of Other Risk Factors and Confounding Factors

Participants completed a self-administered questionnaire that collected sociodemographic information (sex, age, and educational level), medical history (including hypertension, diabetes mellitus [DM], and prior stroke), drinking and smoking habits, and physical activity status (defined as exercising for ≥30 min at least twice weekly for the past year or longer). Trained researchers reviewed the completed questionnaires to identify any inconsistencies or missing responses. The protein–energy ratio was assessed using an original food frequency questionnaire (FFQ). The FFQ comprised 215 questionnaire items. It assessed weekly intake frequency and portion size (small, medium, or large) for 65 foods and beverages selected from 233 food and beverage items. It also asked about the consumption of stir-fried and deep-fried foods and the frequency of eating fatty and lean meats [18]. Daily intakes of foods and nutrients were estimated from the FFQ responses using the Japanese Standard Tables of Food Composition 2015 (7th revised edition). The validity of these estimates was examined by comparison with dietary records collected by a weighed dietary record method for 4 consecutive days in each season, and Pearson’s correlation coefficients were calculated [19]. Hemoglobin A1c levels were assessed in accordance with the National Glycohemoglobin Standardization Program guidelines. DM was defined according to the 2010 American Diabetes Association criteria as a fasting glucose level ≥ 7.0 mmol/L, a random glucose level ≥ 11.1 mmol/L, hemoglobin A1c ≥ 6.5%, or current antidiabetic medication use [20].

Blood pressure was assessed in triplicate, with measurements taken at intervals of at least 5 min; the mean of the three values was used in the analyses. Hypertension was defined as blood pressure ≥140/90 mmHg or current antihypertensive medication use. Body mass index (BMI, kg/m^2^) was used to assess obesity. Serum high-density lipoprotein (HDL) and low-density lipoprotein (LDL) cholesterol levels were measured enzymatically. Serum high-sensitivity C-reactive protein (hs-CRP) levels were analyzed using the latex agglutination turbidimetric method. Apolipoprotein E (*APOE*) ε4 status was assessed by genotyping rs429358 and rs7412 using a multiplex PCR-based targeted sequencing method, as described previously [21]. Handgrip strength and gait speed were measured in all participants according to a standardized protocol. Handgrip strength was measured twice in each hand with one of four digital dynamometers, and the maximum value was used in the analysis. The devices were T.K.K.5001 and 5401 (Takei Scientific Instruments Co., Ltd., Niigata, Japan); YS (Tsutsumi, Tokyo, Japan); 261-006-05YX (Muranaka Medical Instruments, Osaka, Japan); and T-2177 (TOEI, Saitama, Japan). Individuals with upper-limb pain were excluded from this measurement. Usual gait speed was assessed twice on a 5 m walking course at the participant’s normal pace, and the faster measurement was used for analysis. Those with walking difficulty or fall risk were excluded from this assessment.

### 2.6. Statistical Analyses

Clinical characteristics and FFQ-derived intakes of selected food groups and protein-related dietary indices were compared using the Jonckheere–Terpstra test for continuous variables and the Mantel–Haenszel test for categorical variables. Multivariate-adjusted estimates for TBV/eTIV, HV/eTIV, and WMLV/eTIV with corresponding 95% confidence intervals were obtained using analysis of covariance. Effect sizes were expressed as partial eta-squared (partial *η^2^*). Model 1 was adjusted for sex, age, educational level, and research site. Model 2 was additionally adjusted for the presence of the *APOE* ε4 allele, BMI, DM, hypertension, serum HDL and LDL cholesterol levels, protein–calorie intake ratio, serum hs-CRP, drinking and smoking habits, and regular exercise. Model 2 included *APOE* ε4 and vascular, lifestyle, nutritional, and inflammatory factors potentially associated with both serum albumin levels and brain structural measures. Participants with missing data for any variable included in each model were excluded from the corresponding analysis. No substantial multicollinearity was observed among the variables included in Model 2.

For sensitivity analyses, we conducted multivariable-adjusted analyses using albumin quintiles. Additionally, we performed subgroup analyses stratified by maximum handgrip strength and usual gait speed. Serum albumin levels and frailty-related indicators were dichotomized as follows: serum albumin (<4.2 vs. ≥4.2 g/dL); maximum handgrip strength (<28 kg in men or <18 kg in women vs. higher); and usual gait speed (<1.0 vs. ≥1.0 m/s), with the latter two based on the revised Japanese version of the Cardiovascular Health Study criteria (J-CHS) [22]. These categorizations resulted in 2 × 2 stratifications for each indicator. Multivariable-adjusted estimates for TBV/eTIV, HV/eTIV, and WMLV/eTIV were calculated using Model 2. Heterogeneity across subgroups was assessed by including a multiplicative interaction term between albumin category and each frailty indicator in Model 2, and *p* values for interaction were calculated. All statistical analyses were conducted using SPSS software (version 29; SPSS Inc., Chicago, IL, USA). Statistical significance was set at *p* < 0.05.

## 3. Results

### 3.1. Baseline Characteristics

The median serum albumin level in the study population was 4.3 g/dL (interquartile range [IQR]: 4.1–4.5 g/dL). Table 1 summarizes participant characteristics by serum albumin category. Participants were first divided according to the clinical cutoff of 3.5 g/dL, and those with serum albumin ≥ 3.5 g/dL were further grouped into quartiles. Lower serum albumin levels showed significant associations with older age, lower educational level, higher serum hs-CRP, and lower protein–energy ratio, BMI, maximum handgrip strength, usual gait speed, serum LDL cholesterol, and serum HDL cholesterol. Hypertension, *APOE* ε4 carrier status, and regular exercise also differed significantly across albumin categories, although the pattern was less consistent in the lowest albumin category.

### 3.2. Influence on Brain Volumes

Lower serum albumin levels were significantly associated with lower TBV/eTIV and HV/eTIV after adjustment for sex, age, educational level, and study site (Model 1). These associations remained significant after adjustment for sex, age, educational level, study site, presence of APOE ε4, hypertension, DM, BMI, HDL, and LDL cholesterol levels, protein/calorie intake ratio, hs-CRP, drinking and smoking habits, and regular exercise (Model 2) (*p* for trend <0.001; partial *η^2^* = 0.005 for both TBV/eTIV and HV/eTIV). However, there was no evidence of a significant association between serum albumin levels and WMLV/eTIV in either Model 1 or Model 2 (*p* for trend = 0.58 and 0.24, respectively; partial *η^2^* = 0.000 and 0.001, respectively) (Table 2).

### 3.3. Sensitivity Analysis

In the sensitivity analysis, serum albumin was categorized into quintiles, and baseline characteristics across these quintiles are summarized in Supplementary Table S2. The overall patterns were largely consistent with those in the primary analysis. As shown in Supplementary Table S3, TBV/eTIV and HV/eTIV decreased significantly with lower quintile levels of serum albumin in both Model 1 and Model 2 (*p* for trend <0.001; partial *η^2^* = 0.005 for both TBV/eTIV and HV/eTIV). There was no significant association between serum albumin levels and WMLV/eTIV (*p* for trend = 0.48 and 0.28, respectively; partial *η^2^* = 0.000 and 0.001, respectively).

### 3.4. Subgroup Analysis

**Figure 2:**
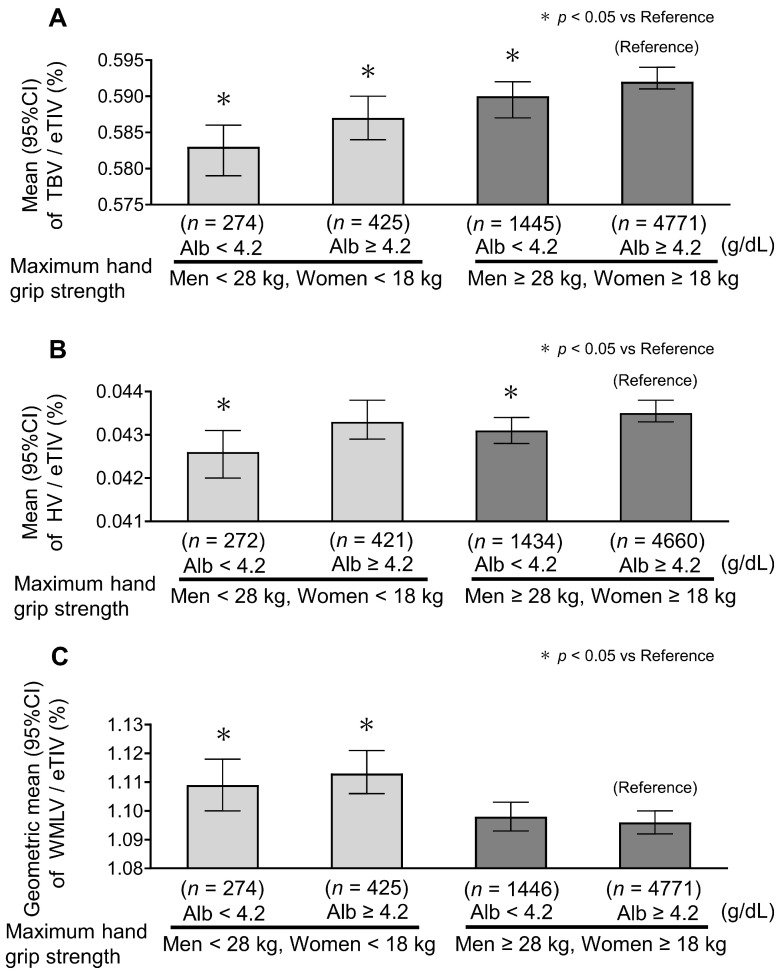
Model 2-adjusted subgroup analysis of total brain volume, hippocampal volume, and white matter lesion volume by serum albumin level and maximum handgrip strength. Serum albumin is categorized as <4.2 vs. ≥4.2 g/dL, with 4.2 g/dL corresponding to the boundary between the primary albumin categories. Maximum handgrip strength was categorized as <28 kg in men, <18 kg in women vs. men ≥28 kg, and women ≥18 kg, based on the revised J-CHS criteria. (**A**) TBV/eTIV. Lower values tended to be observed in the lower albumin group and in participants with reduced maximum handgrip strength. Bars represent the mean, and error bars indicate the 95% confidence intervals. (**B**) HV/eTIV. Lower values tended to be observed in the lower albumin group, even among participants with normal maximum handgrip strength. Bars represent the mean, and error bars indicate the 95% confidence intervals. (**C**) WMLV/eTIV. Higher values tended to be observed mainly in participants with reduced maximum handgrip strength, with limited differences according to albumin level. Bars represent the geometric mean, and error bars indicate the 95% confidence intervals. Model 2 is adjusted for sex, age, education levels, research site, hypertension, diabetes mellitus, body mass index levels, serum low-density lipoprotein and high-density lipoprotein cholesterol levels, the presence of the apolipoprotein E ε4 allele, protein–calorie intake ratio, high-sensitivity CRP, smoking and drinking habits, and regular exercise. Abbreviations: Alb, albumin; TBV, total brain volume; HV, hippocampal volume; WMLV, white matter lesion volume; eTIV, estimated total intracranial volume.

**Figure 3:**
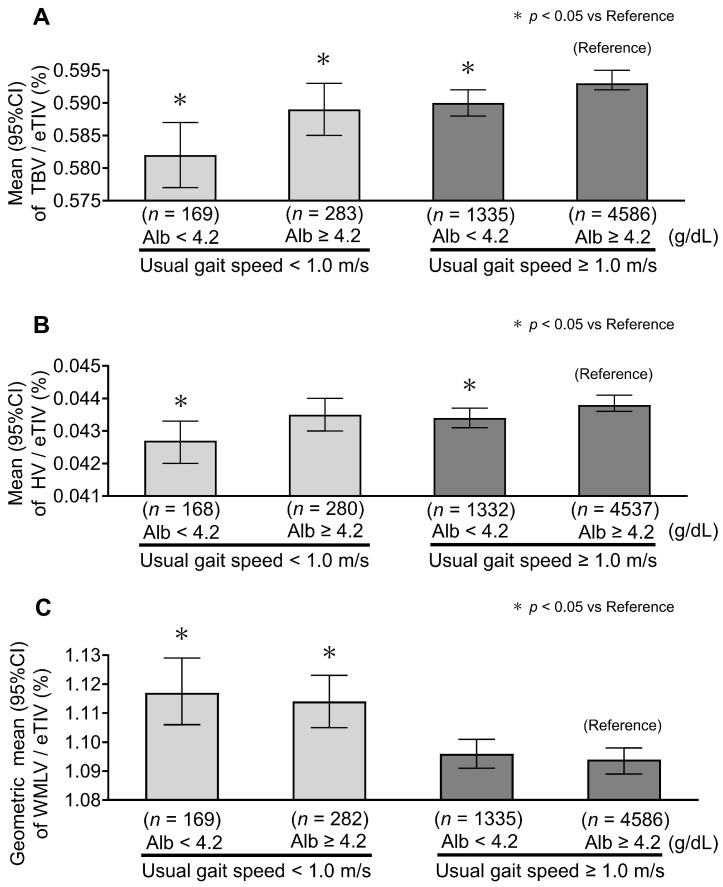
Model 2-adjusted subgroup analysis of total brain volume, hippocampal volume, and white matter lesion volume by serum albumin level and usual gait speed. Serum albumin is categorized as <4.2 vs. ≥4.2 g/dL, with 4.2 g/dL corresponding to the boundary between the primary albumin categories. Usual gait speed is categorized as <1.0 vs. ≥1.0 m/s, based on the revised J-CHS criteria. (**A**) TBV/eTIV. Lower values tended to be observed in the lower albumin group and in participants with slower usual gait speed. Bars represent the mean, and error bars indicate the 95% confidence intervals. (**B**) HV/eTIV. Lower values tended to be observed in the lower albumin group, even among participants with usual gait speed ≥1.0 m/s. Bars represent the mean, and error bars indicate the 95% confidence intervals. (**C**) WMLV/eTIV. Higher values tended to be observed mainly in participants with slower usual gait speed, with limited differences according to albumin level. Bars represent the geometric mean, and error bars indicate the 95% confidence intervals. Model 2 is adjusted for sex, age, education levels, research site, hypertension, diabetes mellitus, body mass index levels, serum low-density lipoprotein and high-density lipoprotein cholesterol levels, the presence of the apolipoprotein E ε4 allele, protein–calorie intake ratio, high-sensitivity CRP, smoking and drinking habits, and regular exercise. Abbreviations: Alb, albumin; TBV, total brain volume; HV, hippocampal volume; WMLV, white matter lesion volume; eTIV, estimated total intracranial volume.

In subgroup analyses, Model 2-adjusted estimates showed no significant interaction between serum albumin levels and either maximum handgrip strength (*p* for interaction = 0.443 for TBV/eTIV, 0.280 for HV/eTIV, and 0.252 for WMLV/eTIV) or usual gait speed (*p* for interaction = 0.210 for TBV/eTIV, 0.364 for HV/eTIV, and 0.856 for WMLV/eTIV). For TBV/eTIV, lower values tended to be observed in participants with lower albumin levels and in those with reduced maximum handgrip strength or slower gait speed (Figure 2A and Figure 3A). However, among participants with normal maximum handgrip strength or usual gait speed, those with lower albumin levels (<4.2 g/dL) tended to have smaller HV/eTIV (Figure 2B and Figure 3B). WMLV/eTIV values were predominantly higher in groups with reduced handgrip strength or slower gait speed, and their association with albumin levels was limited (Figure 2C and Figure 3C). Exact Model 2-adjusted estimates and 95% confidence intervals for the subgroup analyses are provided in Supplementary Tables S4 and S5.

### 3.5. FFQ-Derived Dietary Intakes Across Serum Albumin Categories

As can be seen in Supplementary Table S6, several FFQ-derived protein-related dietary indices differed according to serum albumin levels. Intake of soy and soy products, natto, red fish, yogurt, and cheese was significantly positively associated with increases in serum albumin levels, whereas no significant associations were observed for total protein, fish and shellfish, meat, dairy products, or milk.

## 4. Discussion

This cross-sectional study demonstrated significant associations between lower serum albumin levels and smaller TBV/eTIV and HV/eTIV in cognitively normal, community-dwelling older Japanese adults, despite the small effect sizes. Sensitivity analyses confirmed the consistency of the results. Subgroup analyses further suggested that participants with preserved physical function (normal handgrip strength or usual gait speed) and lower serum albumin levels tended to have smaller HV/eTIV, although no statistically significant interaction was observed.

Several epidemiological studies have reported that hypoalbuminemia is associated with cognitive decline and an increased risk of dementia [2,3,4,5,6]. A study including approximately 1750 adults aged ≥65 years in England demonstrated that lower serum albumin levels were independently associated with the risk of cognitive impairment [4]. In Korean older adults, chronic hypoalbuminemia was strongly associated with lower Mini-Mental State Examination (MMSE) scores [5]. Recent machine learning studies using routine blood test data have reported an association between lower serum albumin levels and lower MMSE scores in individuals aged ≥65 years, along with improved predictive accuracy when albumin is included in the model [6]. Furthermore, a prospective study using UK Biobank data suggested that elevated serum albumin levels were associated with a reduced risk of dementia onset, indicating that serum albumin may play a protective role against dementia [23]. In very old people with Alzheimer’s disease (AD), lower albumin levels were significantly associated with lower predictive MMSE scores and higher Clinical Dementia Rating scores [24]. We found that lower serum albumin levels were associated with smaller brain volume, consistent with previous reports linking serum albumin levels to cognitive outcomes. Given the close association between hippocampal atrophy and AD risk, serum albumin may be associated with brain changes occurring in dementia. Although this study was cross-sectional, longitudinal MRI studies have shown that a smaller hippocampal volume is associated with increased dementia risk [25]. Recent multimodal neuroimaging findings further suggest that hippocampal alterations reflect stage-specific pathways in AD progression [26]. Together, these findings help contextualize the observed association between lower serum albumin levels and smaller hippocampal volume.

No significant association was observed between serum albumin levels and WMLV/eTIV in this study, whereas previous research has reported that hypoalbuminemia is associated with increased periventricular white matter lesions [11]. However, that association was non-linear and confined to the highest albumin quartile. In addition, white matter lesions in the prior study were assessed categorically using a modified Fazekas scale rather than as continuous lesion volume normalized to intracranial volume. These methodological differences may partly explain this discrepancy.

Albumin is also a nutritional biomarker closely associated with frailty. In older adults in the United States, lower Geriatric Nutritional Risk Index (GNRI) scores have been associated with cognitive decline [27]. A systematic review and meta-analysis of adults after stroke found that low GNRI scores were significantly associated with the onset of cognitive impairment [28]. Additionally, low albumin levels have been associated with mild cognitive impairment and frailty, indicating that nutritional status influences both neurocognitive and physical functions [29]. A systematic review and meta-analysis in adults aged ≥60 years found that serum albumin levels were inversely associated with frailty and sarcopenia, supporting the role of serum albumin as a biomarker of malnutrition [30]. Frailty has also been associated with structural brain abnormalities in older adults without cognitive impairment [13,31]. Therefore, frailty is important in the present study not only as a physical performance metric but also as a framework for interpreting the association between serum albumin and brain structure. Moreover, physical decline correlates with brain structure and cognitive function. A decline in gait speed and handgrip strength has been associated with early cognitive decline [32,33], while reduced gait speed has been linked to hippocampal atrophy, lower gray matter volume, cortical thickness, and increased white matter lesions [12,34,35]. Similar associations have been reported for handgrip strength decline [36,37]. Reverse causation should also be considered. Although lower serum albumin may reflect poorer nutritional or systemic status associated with smaller brain volumes, pre-existing brain atrophy may also contribute to lower serum albumin through appetite disturbance or abnormal eating behavior. Longitudinal data suggest that lower baseline brain structure may precede appetite disturbance, and appetite loss in older adults has been associated with malnutrition [38,39].

The dietary analysis provides additional context for this interpretation. Higher serum albumin levels were not accompanied by a significantly different total protein intake but were more often observed in participants with higher intake of specific protein-containing foods, particularly soy and soy products, natto, red fish, yogurt, and cheese. These findings suggest that the food sources of dietary protein, rather than total protein intake, may be relevant to serum albumin status. Previous evidence suggests that certain nutritional substances may promote general well-being even in healthy individuals, supporting continued interest in modifiable nutritional factors in aging research [40].

Subgroup analyses in this study confirmed trends consistent with previous research but also revealed different association patterns for TBV/eTIV and HV/eTIV. TBV/eTIV was reduced even in the high serum albumin group with poor frailty indicators. In contrast, HV/eTIV was reduced in the group with favorable frailty indicators. Notably, HV/eTIV reduction was observed in the low serum albumin group with favorable frailty indicators (handgrip strength and usual gait speed). This suggests that the associations of frailty indicators and serum albumin may differ between TBV/eTIV and HV/eTIV. In particular, it can be reflected by the observed association between lower albumin levels and smaller HV/eTIV even among participants with favorable frailty indicators. Therefore, serum albumin may reflect frailty-related nutritional status, while its association with hippocampal volume may not be fully explained by frailty indicators. However, no significant interaction was observed, precluding the conclusion that frailty and albumin mutually modify each other’s effects.

Several biological pathways may help explain the observed association. Albumin binds circulating amyloid-β and contributes to its peripheral sequestration [41,42]. In cognitively normal older adults, lower serum albumin has been associated with greater cerebral amyloid-β deposition [43]. Accordingly, albumin replacement or plasma exchange has shown beneficial effects in a clinical trial [44] and reduced amyloid plaque burden in a mouse model [45]. Oxidative stress may also be relevant, as albumin has been reported to reduce oxidative stress and neuronal apoptosis in an experimental study [9]. More broadly, serum albumin has also been linked to several neurodegenerative disorders [46,47,48,49], suggesting that albumin-related pathways may not be limited to AD pathology. Together, these findings provide a biological context for the association between serum albumin and brain structural measures in older adults.

This study has some limitations. First, the sample size in the low-albumin group was small (*n* = 31), potentially affecting the results. In the subgroup analyses, further stratification of the already small low-albumin group may have limited statistical power to detect interaction effects. Therefore, the absence of significant interaction should be interpreted cautiously. To address this limitation, we performed sensitivity analyses, and the results were consistent with the main findings. Although the effect sizes were small, suggesting limited clinical relevance, we believe our findings remain meaningful for several reasons. Our study provides novel evidence linking serum albumin levels with brain structural features, contributing to a better understanding of the underlying biological mechanisms. In addition, the robustness of our findings is supported by the overall sample size and careful adjustment for potential confounders. Taken together, while the clinical impact for an individual may be modest, our results offer valuable insights and a foundation for future longitudinal and interventional studies. Second, because of the cross-sectional study design, establishing causality is difficult. Third, nutritional status was not comprehensively assessed. Serum albumin may reflect both nutritional status and broader physiological dysregulation related to common chronic conditions in older adults. Its levels may be influenced by additional factors such as liver function, hydration status, and inflammation. Fourth, frailty was not formally assessed in this analysis. Subgroup analyses relied on maximum handgrip strength and usual gait speed as frailty-related indicators. Fifth, although MRI acquisition was standardized across sites, some inter-site variability may have remained. Selection bias is also possible, as participants who underwent MRI may have been healthier than the source population. In addition, because the study included older Japanese adults, the generalizability of the findings to other ethnic populations or age groups may be limited. However, the use of a large sample size, adjustment for numerous confounding factors, and exclusion of participants with dementia and mild cognitive impairment strengthen the validity of the findings.

## 5. Conclusions

This study shows that lower serum albumin levels are associated with smaller TBV/eTIV and HV/eTIV in cognitively normal older Japanese adults. Our findings also suggest that consumption of protein-rich foods, such as soy, soy products, natto, red fish, yogurt, and cheese, may be associated with better nutritional status among community-dwelling older adults. Because serum albumin testing is routinely available in clinical practice, serum albumin may be a clinically accessible marker related to brain structural features in older adults. Future longitudinal studies and basic research are needed to further clarify the underlying pathophysiological pathways and evaluate potential intervention strategies.

## Figures and Tables

**Table 1:** Clinical characteristics according to serum albumin levels.

Variables	Serum Albumin (g/dL)					*p* for Trend
	<3.5	3.5–4.1	4.2	4.3–4.5	≥4.6	
	(*n* = 31)	(*n* = 1808)	(*n* = 859)	(*n* = 2757)	(*n* = 1811)	
Age, years	76.0 (72.0–83.0)	73.0 (69.0–78.0)	71.0 (68.0–76.0)	70.0 (67.0–75.0)	70.0 (67.0–74.0)	<0.001 *
Women, %	45.2	57.5	59.5	59.8	60.5	0.033 *
Hypertension, %	77.4	68.5	68.5	72.6	76.6	<0.001 *
Diabetes mellitus, %	36.7	15.3	13.7	16.2	16.8	0.262
Serum HDL-chol, mg/dL	52.0 (42.0–60.0)	58.0 (48.0–69.0)	60.0 (50.0–72.0)	62.0 (51.0–73.0)	65.0 (54.0–77.0)	<0.001 *
Serum LDL-chol, mg/dL	98.0 (79.0–109.0)	110.0 (91.0–129.0)	114.0 (96.0–135.0)	119.0 (99.0–138.0)	123.0 (103.0–143.75)	<0.001 *
Serum hs-CRP, mg/dL	0.11 (0.05–0.50)	0.06 (0.03–0.14)	0.05 (0.02–0.10)	0.05 (0.02–0.09)	0.04 (0.02–0.08)	<0.001 *
Body mass index, kg/m^2^	23.07 (20.42–24.80)	23.23 (21.23–25.62)	23.26 (21.25–25.40)	23.20 (21.23–25.29)	22.93 (21.02–24.91)	<0.001 *
*APOE*4 ε4, present, %	17.9	15.2	17.9	19.5	17.9	0.011 *
Education ≤ 9 years, %	41.9	30.1	27.6	22.7	22.3	<0.001 *
Current alcohol intakes, %	36.7	42.9	44.0	44.3	46.0	0.025 *
Current smoking, %	13.3	8.9	8.8	6.9	8.4	0.093
Regular exercise, %	45.2	41.4	42.9	43.7	48.1	<0.001 *
Protein/total calorie intake ratio	15.48 (14.60–17.38)	15.52 (13.72–17.20)	15.86 (13.98–17.58)	15.72 (13.94–17.53)	15.72 (13.98–17.64)	0.004 *
Maximum handgrip strength, kg	23.50 (18.30–32.40)	25.30 (21.0–33.50)	26.0 (21.70–33.75)	26.20 (22.0–34.90)	26.90 (22.80–35.0)	<0.001 *
Usual gait speed, m/s	1.16 (0.98–1.39)	1.32 (1.16–1.47)	1.35 (1.19–1.50)	1.39 (1.22–1.53)	1.43 (1.27–1.56)	<0.001 *

Abbreviations: *APOE*, apolipoprotein E; HDL-chol, high-density lipoprotein cholesterol; LDL-chol, low-density lipoprotein cholesterol; hs-CRP, high-sensitivity C-reactive protein; IQR, interquartile range. * *p* for trend < 0.05. Values are shown as median (interquartile range) for continuous variables or percentages for categorical variables.

**Table 2:** Multivariable-adjusted mean values of the volumes of total brain volume, hippocampus volume, and white matter lesion volume according to serum albumin levels.

	Serum Albumin (g/dL)					*p* for Trend	Partial *η*^2^
	<3.5	3.5–4.1	4.2	4.3–4.5	≥4.6		
Total brain volume/eTIV (%)							
Model 1	0.571 (0.560–0.582) (*n* = 28)	0.592 (0.591–0.592) (*n* = 1781)	0.592 (0.590–0.594) (*n* = 838)	0.594 (0.592–0.595) (*n* = 2696)	0.596 (0.594–0.598) (*n* = 1784)	<0.001 *	0.006
Model 2	0.571 (0.560–0.582) (*n* = 26)	0.588 (0.586–0.591) (*n* = 1709)	0.590 (0.587–0.592) (*n* = 816)	0.591 (0.590–0.593) (*n* = 2613)	0.593 (0.591–0.596) (*n* = 1729)	<0.001 *	0.005
Hippocampal volume/eTIV (%)							
Model 1	0.0420 (0.0405–0.0436) (*n* = 28)	0.0431 (0.0429–0.0434) (*n* = 1768)	0.0432 (0.0429–0.0435) (*n* = 830)	0.0436 (0.0435–0.0438) (*n* = 2664)	0.0439 (0.0437–0.0442) (*n* = 1765)	<0.001 *	0.006
Model 2	0.0419 (0.0403–0.0435) (*n* = 26)	0.0430 (0.0428–0.0433) (*n* = 1696)	0.0431 (0.0428–0.0435) (*n* = 808)	0.0436 (0.0433–0.0438) (*n* = 2584)	0.0439 (0.0436–0.0442) (*n* = 1711)	<0.001 *	0.005
White matter lesions volume /eTIV (%)							
Model 1	1.102 (1.076–1.128) (*n* = 28)	1.099 (1.095–1.103) (*n* = 1782)	1.101 (1.096–1.106) (*n* = 838)	1.097 (1.093–1.10) (*n* = 2964)	1.098 (1.094–1.102) (*n* = 1785)	0.58	0.000
Model 2	1.104 (1.077–1.131) (*n* = 26)	1.101 (1.096–1.105) (*n* = 1710)	1.103 (1.097–1.109) (*n* = 817)	1.097 (1.093–1.101) (*n* = 2611)	1.098 (1.093–1.103) (*n* = 1730)	0.24	0.001

Abbreviations: eTIV, estimated total intracranial volume; HV, hippocampal volume; TBV, total brain volume; * *p* for trend <0.05. TBV/eTIV and HV/eTIV values are shown as a mean (95% confidence interval). WMLV/eTIV values are shown as a geometric mean (95% confidence interval). Model 1 is adjusted for sex, age, education levels, and research site. Model 2 is adjusted for sex, age, education levels, research site, hypertension, diabetes mellitus, body mass index levels, serum low-density lipoprotein and high-density lipoprotein cholesterol levels, the presence of the apolipoprotein E ε4 allele, protein–calorie intake ratio, serum high-sensitivity C-reactive protein, smoking and drinking habits, and regular exercise.

## Data Availability

The data used and/or analyzed in this study are not publicly available because the informed consent provided by the participants does not permit unrestricted public disclosure. However, data are available from the authors upon reasonable request and with permission from the steering committee of JPSC-AD and the Japan Agency for Medical Research and Development.

## Supplementary Materials

The following supporting information can be downloaded at: https://www.mdpi.com/article/10.3390/nu18101520/s1, Table S1: Methods of serum albumin measurement; Table S2: Clinical characteristics according to quintiles of serum albumin level; Table S3: Sensitivity analysis of multivariable-adjusted mean total brain volume, hippocampal volume, and white matter lesion volume according to quintiles of serum albumin levels; Table S4: Model 2-adjusted subgroup analysis of total brain volume, hippocampal volume, and white matter lesion volume by serum albumin level and maximum handgrip strength; Table S5: Model 2-adjusted subgroup analysis of total brain volume, hippocampal volume, and white matter lesion volume by serum albumin level and usual gait speed; Table S6: FFQ-derived intakes of protein-related indices according to serum albumin levels.

## Abbreviations

The following abbreviations are used in this manuscript:

AD	Alzheimer’s disease
BMI	Body mass index
DM	Diabetes mellitus
FFQ	Food frequency questionnaire
GNRI	Geriatric Nutritional Risk Index
HDL	High-density lipoprotein
HV	Hippocampal volume
J-CHS	Japanese version of the Cardiovascular Health Study criteria
LDL	Low-density lipoprotein
MMSE	Mini-Mental State Examination
MRI	Magnetic resonance imaging
TBV	Total brain volume
WMLV	White matter lesion volume
