# Supplementary material for: Serum Albumin Levels Are Associated with Total Brain and Hippocampal Volume but Not with White Matter Lesion Volume in Older Japanese Adults Without Cognitive Decline: A Cross-Sectional Study
Source: Nutrients. 2026 May 10;18(10):1520. doi: 10.3390/nu18101520 (PMC13209699; doi:10.3390/nu18101520)
Supplement: Supplementary file 1 [file nutrients-18-01520-s001.zip › nutrients-4211010-supplementary.pdf]

Supplementary Table S1. Methods of serum albumin measurement

Reagent	Reagent name	Iatro ALB
	Product number	13E1X80073000031
	Manufacturing company	Nittobo Medical, Tokyo, Japan
Measuring instrument	Measuring instrument	Hitachi 7700 until December 23, 2017; Hitachi 008α and JEOL JCA-BM8060 from December 25, 2017
	Manufacturing company	Hitachi High-Technologies Corporation, Tokyo, Japan
	Measuring method	Modified bromocresol purple method
Standardization	Reference material for calibration	IFCC international reference material for plasma proteins (ERM-DA470)

Supplementary Table S2. Clinical characteristics according to quintiles of serum albumin level.

**Variables**	**Serum albumin (g/dL)**					***p* for trend**
	**<4.1 (*n* = 1129)**	**4.1–4.2 (*n* = 1569)**	**4.3 (*n* = 992)**	**4.4–4.5 (*n* = 1765)**	**≥4.6 (*n* = 1811)**	
Age, years	74.0 (69.0–79.0)	72.0 (68.0–77.0)	71.0 (67.0–75.0)	70.0 (67.0–75.0)	70.0 (67.0–74.0)	<0.001*
Women, %	55.9	59.5	60.6	59.4	60.5	0.043*
Hypertension, %	69.4	68.0	69.4	74.4	76.6	<0.001*
Diabetes mellitus, %	16.5	14.0	15.4	16.7	16.8	0.151
Serum HDL-chol, mg/dl	57.0 (48.0–69.0)	60.0 (49.0–71.0)	62.0 (52.0–73.0)	62.0 (52.0–73.0)	65.0 (54.0–77.0)	<0.001*
Serum LDL-chol, mg/dl	109.0 (90–129)	113.0 (95.0–133.0)	119.0 (99.9–138.0)	119.0 (99.0–139.0)	123.0 (103–143.75)	<0.001*
Serum hs-CRP, mg/dl	0.07 (0.03–0.16)	0.05 (0.03–0.11)	0.05 (0.02–0.09)	0.04 (0.02–0.09)	0.04 (0.02–0.08)	<0.001*
Body mass index, kg/m²	23.22 (21.13–25.61)	23.26 (21.32–25.53)	23.25 (21.16–25.50)	23.18 (21.26–25.22)	22.93 (21.02–24.92)	0.001*
*APOE*4 ε4, present, %	16.1	16.1	18.9	19.8	17.9	0.029*
Education ≤9 years, %	31.9	27.7	23.8	22.1	22.3	<0.001*
Current alcohol intakes, %	42.8	43.5	42.4	45.4	46.0	0.036*
Current smoking, %	10.1	8.1	8.1	9.1	6.9	0.076
Regular exercise, %	41.2	42.4	42.1	44.5	48.1	<0.001*
Protein/total calorie intake ratio	15.4 (13.53–17.16)	15.78 (13.94–17.46)	15.74 (14.12–17.67)	15.72 (13.88–17.47)	15.72 (13.98–17.64)	0.008*
Maximum handgrip strength, kg	25.0 (20.70–33.45)	25.92 (21.50–34.00)	26.0 (22.0–34.70)	26.4 (22.10–35.0)	26.90 (22.80–35.0)	<0.001*
Usual gait speed, m/s	1.29 (1.12–1.47)	1.35 (1.19–1.49)	1.37 (1.21–1.52)	1.39 (1.25–1.54)	1.43 (1.27–1.56)	<0.001*

Abbreviations: *APOE*; Apolipoprotein E, HDL-chol; High-density lipoprotein cholesterol, LDL-chol; low-density lipoprotein cholesterol, hs-CRP; high-sensitivity C reactive protein, IQR; interquartile range. ∗*p* for trend < 0.05.
Values are shown as median (interquartile range) for continuous variables or percentage for categorical variables.

Supplementary Table S3. Sensitivity analysis of multivariable-adjusted mean total brain volume, hippocampal volume, and white matter lesion volume according to quintiles of serum albumin levels.

	**Serum albumin (g/dL)**					***p* for trend**	**partial *η*2**
	**<4.1**	**4.1–4.2**	**4.3**	**4.4–4.5**	**≥4.6**		
			Total brain volume / eTIV (%)				
Model 1	0.589 (0.587–0.591) (*n* = 1108)	0.592 (0.591–0.594) (*n* = 1539)	0.592 (0.590–0.594) (*n* = 966)	0.595 (0.593–0.596) (*n* = 1730)	0.596 (0.594–0.598) (*n* = 1784)	<0.001*	0.006
Model 2	0.587 (0.585–0.589) (*n* = 1057)	0.590 (0.588–0.592) (*n* = 1494)	0.590 (0.588–0.592) (*n* = 940)	0.592 (0.590–0.594) (*n* = 1673)	0.593 (0.591–0.596) (*n* = 1729)	<0.001*	0.005
			Hippocampal volume / eTIV (%)				
Model 1	0.0430 (0.0427–0.0433) (*n* = 1102)	0.0433 (0.0430–0.0435) (*n* = 1524)	0.0436 (0.0433–0.0439) (*n* = 958)	0.0437 (0.0435–0.0439) (*n* = 1706)	0.0440 (0.0437–0.0442) (*n* = 1765)	<0.001*	0.006
Model 2	0.0429 (0.0426–0.0432) (*n* = 1051)	0.0432 (0.0429–0.0435) (*n* = 1479)	0.0435 (0.0432–0.0438) (*n* = 933)	0.0436 (0.0433–0.0439) (*n* = 1651)	0.0439 (0.0436–0.0442) (*n* = 1711)	<0.001*	0.005
			White matter hyperintensity / eTIV (%)				
Model 1	1.101 (1.096–1.106) (*n* = 1108)	1.099 (1.095–1.103) (*n* = 1540)	1.095 (1.091–1.10) (*n* = 966)	1.097 (1.093–1.101) (*n* = 1728)	1.098 (1.094–1.102) (*n* = 1785)	0.48	0.000
Model 2	1.102 (1.096–1.107) (*n* = 1057)	1.101 (1.096–1.106) (*n* = 1496)	1.097 (1.091–1.103) (*n* = 940)	1.097 (1.092–1.102) (*n* = 1671)	1.098 (1.093–1.103) (*n* = 1730)	0.28	0.001

Abbreviations: eTIV; estimated total intracranial volume.

∗*p* for trend < 0.05.

TBV/eTIV and HV/eTIV values were shown as a mean (95% confidence interval).

WMLV/eTIV values were shown as a geometric mean (95% confidence interval).

Model 1 was adjusted for sex, age, education levels and research site.

Model 2 was adjusted for age, sex, research site, education levels, hypertension, diabetes mellitus, body mass index levels, serum low-density lipoprotein and high-density lipoprotein cholesterol levels, the presence of Apolipoprotein E ε4 allele, protein/calorie intake ratio, serum high-sensitivity C reactive protein and smoking and drinking habits, regular exercise.

Supplementary Table S4. Model 2-adjusted subgroup analysis of total brain volume, hippocampal volume, and white matter lesion volume by serum albumin level and maximum handgrip strength.

Serum albumin (g/dL)				*p* for interaction
**<4.2**	**≥4.2**	**<4.2**	**≥4.2**	
**Men<28kg, Women<18kg**		**Men≥28kg, Women≥18kg**		
Total brain volume / eTIV (%)				
0.583 (0.579-0.586)<br>(*n* = 274)	0.587 (0.584-0.590)<br>(*n* = 425)	0.590 (0.587-0.592)<br>(*n* = 1445)	0.592 (0.591-0.594)<br>(*n* = 4771)	0.443
Hippocampal volume / eTIV (%)				
0.0426 (0.0421-0.0431)<br>(*n* = 272)	0.0434 (0.0429-0.0438)<br>(*n* = 421)	0.0432 (0.0429-0.0435)<br>(*n* = 1434)	0.0435 (0.0433-0.0438)<br>(*n* = 4660)	0.28
White matter hyperintensity / eTIV (%)				
1.109 (1.10-1.118)<br>(*n* = 274)	1.113 (1.106-1.121)<br>(*n* = 425)	1.098 (1.093-1.103)<br>(*n* = 1446)	1.096 (1.092-1.10)<br>(*n* = 4771)	0.252

Abbreviations: eTIV; estimated total intracranial volume, HV; hippocampal volume, TBV; total brain volume, WMLV; white matter lesions volume.

$*p$ for interaction < 0.05.

TBV/eTIV and HV/eTIV values were shown as a mean (95% confidence interval).

WMLV/eTIV values were shown as a geometric mean (95% confidence interval).

Model 2 was adjusted for age, sex, research site, education levels, hypertension, diabetes mellitus, body mass index levels, serum low-density lipoprotein and high-density lipoprotein cholesterol levels, the presence of Apolipoprotein E ε4 allele, protein/calorie intake ratio, serum high-sensitivity C reactive protein and smoking and drinking habits, regular exercise.

Supplementary Table S5. Model 2-adjusted subgroup analysis of total brain volume, hippocampal volume, and white matter lesion volume by serum albumin level and usual gait speed.

**Serum albumin (g/dL)**				***p* for interaction**
**<4.2**	**≥4.2**	**<4.2**	**≥4.2**	
**Usual gait speed<1m/s**		**Usual gait speed≥1m/s**		
Total brain volume / eTIV (%)				
0.582 (0.577-0.587) (n = 169)	0.589 (0.585-0.593) (n = 283)	0.590 (0.588-0.592) (n = 1335)	0.593 (0.592-0.595) (n = 4586)	0.210
Hippocampal volume / eTIV (%)				
0.0427 (0.0420-0.0433) (n = 168)	0.0435 (0.0430-0.0440) (n = 280)	0.0434 (0.0431-0.0437) (n = 1332)	0.0438 (0.0436-0.0441) (n = 4537)	0.364
White matter hyperintensity / eTIV (%)				
1.117 (1.106-1.129) (n = 169)	1.114 (1.105-1.123) (n = 282)	1.096 (1.091-1.101) (n = 1335)	1.094 (1.090-1.098) (n = 4586)	0.856

Abbreviations: eTIV; estimated total intracranial volume, HV; hippocampal volume, TBV; total brain volume, WMLV; white matter lesions volume.

$*p$ for interaction < 0.05.

TBV/eTIV and HV/eTIV values were shown as a mean (95% confidence interval).

WMLV/eTIV values were shown as a geometric mean (95% confidence interval).

Model 2 was adjusted for age, sex, research site, education levels, hypertension, diabetes mellitus, body mass index levels, serum low-density lipoprotein and high-density lipoprotein cholesterol levels, the presence of Apolipoprotein E ε4 allele, protein/calorie intake ratio, serum high-sensitivity C reactive protein and smoking and drinking habits, regular exercise.

Supplementary Table S6. FFQ-derived intakes of protein-related indices according to serum albumin levels.						
**Variables**	**Serum albumin (g/dL)**					***p* for trend**
	**<3.5 (*n* = 31)**	**3.5–4.1 (*n* = 1808)**	**4.2 (*n* = 859)**	**4.3–4.5 (*n* = 2757)**	**≥4.6 (*n* = 1811)**	
Protein/total calorie intake ratio	15.48 (14.60–17.38)	15.52 (13.72–17.20)	15.86 (13.98–17.58)	15.72 (13.94–17.53)	15.72 (13.98–17.64)	0.004*
Total protein, g	78.59 (62.06-83.97)	69.86 (58.30-83.28)	71.47 (60.25-84.79)	70.80 (58.83-84.62)	70.32 (58.16-84.18)	0.260
Soy & Soy Products, g	5.7 (0.0–28.57)	11.43 (2.86–22.86)	11.43 (2.86–28.57)	17.14 (5.71–28.57)	17.14 (5.71–28.57)	<0.001*
Tofu, g	77.10 (25.70-154.3)	77.10 (51.40-102.9)	77.10 (51.40-102.9)	77.10 (51.40-102.9)	77.10 (51.40-102.9)	0.022*
Natto, g	5.70 (0.0-28.60)	11.40 (0.0-22.90))	11.40 (2.90-22.90)	14.30 (5.70-28.60)	17.10 (5.70-28.60)	<0.001*
Fish & Shellfish, g	81.43 (62.86–122.9)	74.29 (48.57–102.9)	77.14 (51.43–106.4)	74.29 (51.43–104.3)	74.29 (50.0–102.9)	0.242
White Fish, g	28.60 (14.30–28.60)	14.30 (14.30-28.60))	14.30 (14.30-28.60)	14.30 (14.30-28.60)	14.30 (14.30-28.60)	0.966
Blue Fish, g	35.70 (14.30–42.90)	28.60 (14.30-42.90)	28.60 (14.30-42.90)	28.60 (14.30-42.90)	28.60 (14.30-42.90)	0.103
Red Fish, g	14.30 (0.0–28.60)	14.30 (0.0-28.60)	14.30 (0.0-28.60)	14.30 (7.10-28.60)	14.30 (7.10-28.60)	<0.001*
Meat, g	61.43 (42.86–77.14)	57.14 (37.14–81.43)	60.0 (38.57–83.93)	60.0 (39.29–81.43)	57.14 (38.57–79.64)	0.199
Beef	11.40 (5.70–17.10)	11.40 (0.0-22.90))	11.40 (5.70–22.90)	11.40 (0.0-17.10)	11.40 (0.0-17.10)	0.009*
Pork	22.90 (11.40–34.30)	22.90 (11.40-34.30)	22.90 (11.40-34.30)	22.90 (11.40-34.30)	22.90 (11.40-34.30)	0.065
Chicken	11.40 (5.70–22.90)	17.10 (11.40-34.30)	22.90 (11.40-34.30)	17.10 (11.40-34.30)	17.10 (11.40-22.90)	0.638
Egg, g	28.57 (21.43–35.71)	25.0 (17.14–42.86)	28.57 (14.29–35.71)	22.86 (14.29–35.71)	21.43 (14.29–35.71)	0.068
Dairy products, g	180.0 (76.29–260.0)	171.4 (88.57–248.6)	168.6 (90.0–250.0)	167.1 (92.86–255.0)	177.1 (95.35–258.6)	0.357
Milk, g	51.40 (0.0-180.0)	45.0 (0.0-154.3)	25.70 (0.0-154.3)	32.10 (0.0-147.9)	38.60 (0.0-143.6)	0.728
Yogurt, g	7.10 (0.0-50.0)	42.90 (0.0-100.0)	42.90 (7.10-100.0)	42.90 (7.10-100.0)	50.0 (14.30-100.0)	<0.001*
Cheese, g	1.40 (0.0-5.70)	1.40 (0.0-5.70)	2.90 (0.0-8.60)	2.90 (0.0-5.70)	2.90 (0.0-8.60)	0.009*

*p for trend < 0.05.

Values are shown as median (interquartile range) for continuous variables or percentage for categorical variables.
